# Ethylene Formation by Catalytic Dehydration of Ethanol with Industrial Considerations

**DOI:** 10.3390/ma6010101

**Published:** 2012-12-28

**Authors:** Denise Fan, Der-Jong Dai, Ho-Shing Wu

**Affiliations:** 1 Department of Chemical and Biomolecular Engineering, University of California Los Angeles, Los Angeles, CA 90095, USA; E-Mail: denise91789@yahoo.com; 2 Department of Chemical Engineering and Materials Science, Yuan Ze University, Chung-Li 32003, Taiwan; E-Mail: chuy2664@gmail.com

**Keywords:** ethanol, dehydration, ethylene, catalyst selectivity, industry, catalyst stability

## Abstract

Ethylene is the primary component in most plastics, making it economically valuable. It is produced primarily by steam-cracking of hydrocarbons, but can alternatively be produced by the dehydration of ethanol, which can be produced from fermentation processes using renewable substrates such as glucose, starch and others. Due to rising oil prices, researchers now look at alternative reactions to produce green ethylene, but the process is far from being as economically competitive as using fossil fuels. Many studies have investigated catalysts and new reaction engineering technologies to increase ethylene yield and to lower reaction temperature, in an effort to make the reaction applicable in industry and most cost-efficient. This paper presents various lab synthesized catalysts, reaction conditions, and reactor technologies that achieved high ethylene yield at reasonable reaction temperatures, and evaluates their practicality in industrial application in comparison with steam-cracking plants. The most promising were found to be a nanoscale catalyst HZSM-5 with 99.7% ethylene selectivity at 240 °C and 630 h lifespan, using a microreactor technology with mechanical vapor recompression, and algae-produced ethanol to make ethylene.

## 1. Introduction

**Figure 1:**
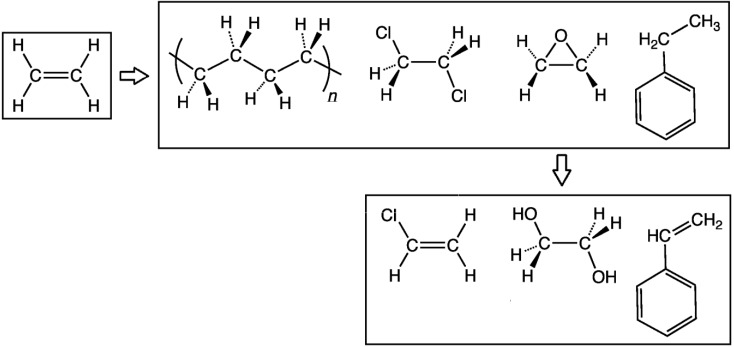
Main uses of ethylene in industry (left to right): polyethylene, ethylene dichloride (precursor to vinyl chloride, below), ethylene oxide (precursor to ethylene glycol, below), and ethylbenzene (precursor to styrene, below).

**Figure 2:**
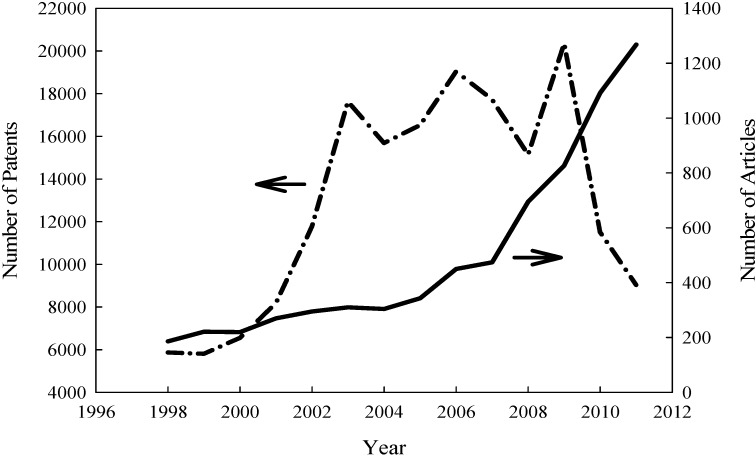
Trend in number of patents (dash-dot line) and publications (solid line) since 1998. Research done on Scopus with key words: ethanol dehydration and ethylene, ethanol dehydration and ethene, ethanol and ethylene production. Total results per type per year for the three searches were summed together.

Ethylene is the most widely produced organic compound in the chemical industry. The large global demand for the compound stems from its various uses as precursors to polymers such as polyethylene, found in most plastics, or surfactant chemicals such as ethylene oxide or ethylene glycol (Figure 1), according to Chemical and Engineering News (2006) [1,2]. The Organization of Economic Cooperation and Development reported that production of ethylene is well over 100 million tons annually, and this market size attracts both industrial companies and scientific researchers alike, as shown by the study history represented graphically in Figure 2 [3]. Figure 2 shows patents and article papers on published year from 1998 to 2011, which is illustrated in the result of the patent, emerged as a rising trend year by year from 1998 to 2005, and dropped to show a rising trend after 2006 and 2008. Of particular interest are alternative methods for synthesizing ethylene, especially with fossil fuel reserves diminishing and, consequently, gas prices and production costs steadily rising. Traditionally, ethylene is produced by steam cracking hydrocarbons, Kniel *et al.* (1980) claims, and this method continues to dominate the industry today [4]. True (2012) reported the top ethylene producing complexes listed in Table 1 (ranked by capacity in tons per year), which are all steam cracking plants, and Conti (2012) reported the capacities of the Braskem and Solvay Indupa ethanol to ethylene plants, while Voegle (2012) reported the capacity of the Dow Chemical plant currently under construction [5,6,7]. However, attention has recently shifted towards green alternatives for manufacturing ethylene, to reduce greenhouse gas emissions and dependency on limited fossil fuels. Leading this green trend is the production of ethylene by catalytic bioethanol dehydration.

**Table 1:** Top industrial ethylene complexes and their locations ranked by capacity (tons of ethylene produced per year).

Company	Location	Ton/year
*Steam-cracking plants*	–	–
Formosa Petrochemical Corporation	Mailiao, Taiwan,	2,935,000
Nova Chemicals Corporation	Joffre, Alberta, Canada	2,811,792
Arabian Petrochemical Company	Jubail, Saudi Arabia	2,250,000
ExxonMobil Chemical Company	Baytown, TX, USA	2,197,000
ChevronPhillips Chemical Company	Sweeny, TX, USA	1,865,000
Dow Chemical Company	Terneuzen, Netherlands	1,800,000
Ineos Olefins & Polymers	Chocolate Bayou, TX, USA	1,752,000
Equistar Chemicals LP	Channelview, TX, USA	1,750,000
Yanbu Petrochemical Company	Yanbu, Saudi Arabia	1,705,000
Equate Petrochemical Company	Shuaiba, Kuwait	1,650,000
*Ethanol to ethylene plants*	–	–
Braskem	Triunfo, Brazil	200,000
Dow Chemical Company	Santa Vitoria, Brazil (under construction)	190,000
Solvay Indupa	Santo Andre, Brazil	60,000

**Figure 3:**
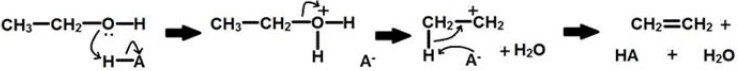
Mechanism for the dehydration of ethanol to ethylene.

In the catalytic dehydration of ethanol to form ethylene, an acid catalyst first protonates the hydroxyl group, which leaves as a water molecule. The conjugate base of the catalyst then deprotonates the methyl group, and the hydrocarbon rearranges into ethylene. This mechanism is depicted in Figure 3. The reaction is endothermic, and because of this, the optimal reaction temperature is fairly high, ranging from 180 °C to 500 °C. Maintaining the reaction temperature constitutes much of the energy cost in industrial application of the reaction, since competing reactions into diethyl ether or acetaldehyde are favored outside of the temperature range and so decrease ethylene yield.

To make ethanol dehydration more industry-friendly, many researchers have investigated different catalysts to increase ethylene yield and lower reaction temperature. Researched catalysts began with alumina and transition metal oxides, but have now expanded to include many modified versions of old catalysts, silicoaluminophosphates (SAPO), HZSM-5 zeolite catalyst, and heteropolyacid catalysts. While current catalysts have achieved much better results than the original ones in terms of yield and reaction temperature, most are still not ready for commercialization. Of the SAPO catalysts studied by Zhang *et al.* (2008), Chen *et al.* (2010), and Wu *et al.* (2011), SAPO-11-4 had the best results with 98.0% ethylene selectivity at 250 °C, but with many catalysts such as modified HZSM-5 and MCM-41 achieving over 99.0% selectivity, SAPO catalysts are not competitive enough [8,9,10]. Likewise, H-mordenites studied by Takahara *et al.* (2005) achieved results like 99.9% ethylene selectivity at 180 °C, but due to the inaccessibility of the catalysts, they are not ideal for commercialization [11]. Bokade *et al.* (2011) studied montmorillonite catalysts, and Zaki (2005) studied manganese oxide and iron oxide modified alumina and silica, but high reaction temperatures of 500 °C for Zaki and low ethylene selectivity for Bokade eliminated those catalysts as possible industrial catalysts [12,13].

The Braskem (Brazil) ethanol to ethylene plant began operation in 2010 and is currently the only plant of its kind at the commercial scale [14]. Although it is considered commercial scale, it produces 200,000 tons of ethylene per year, which pales in comparison with the millions of tons of ethylene capacity that the top steam cracking plants have. In order to make green ethylene plants more competitive, further advancements must be made from Braskem’s first step towards environmentally friendly ethylene production. This review article will discuss the catalysts recently studied that have the potential to be applied in an industrial scale ethanol to ethylene plant competitive with steam cracking plants. In addition, other innovative technologies that should be considered for such a plant are presented. Because other research papers on the dehydration of ethanol to ethylene do not report findings in the context of industry, catalysts and technology in relation to production costs and feasibility are also evaluated.

## 2. Catalysis

### 2.1. Modification of γ-Al_2_O_3_

From the very beginnings of the studies of ethanol dehydration to ethylene, γ-alumina has been used as a catalyst for the reaction. However, due to the high reaction temperature of 450 °C required and the relatively low ethylene yield of 80%, researchers now look to modify the catalyst to reduce the reaction temperature and increase ethylene yield to make it more economically efficient. Phillips Oil Company (USA) utilizes γ-alumina treated with KOH and ZnO/Al_2_O_3_ in its production of ethylene and Halcon SD (USA) has applied MgO-Al_2_O_3_/SiO_2_ based SynDol catalyst in their facilities [15]. Research on more modifications continues, with Doheim *et al.* (2002) achieving 97% ethanol conversion at 300 °C with Na_2_O-doped Mn_2_O_3_/Al_2_O_3_ [16]. Chen *et al.* (2007) investigated the catalytic ability of TiO_2_ modified γ-Al_2_O_3_ [15]. The TiO_2_/γ-Al_2_O_3_ catalyst was prepared by mixing commercial γ-Al_2_O_3_ powder with Na_2_CO_3_ solution, then adding Ti(SO_4_)_2_ and Na_2_CO_3_ solution. SO_4_^2−^ was eliminated by washing and centrifugation; then, the material was dried and calcined to produce TiO_2_/γ-Al_2_O_3_. Tests on the catalytic ability resulted in up to 99.4% ethylene selectivity at 500 °C, significantly higher than the maximum 90.1% ethylene selectivity at 475 °C of γ-Al_2_O_3_ reported by Zhang *et al.* (2008) [10]. Ethanol conversion and ethylene selectivity were found to increase from 75% conversion and 40% selectivity to 100% conversion and 99.4% selectivity with temperature increases from 360 °C to 500 °C. They also decreased from 100% conversion and 97% selectivity to 96% conversion and 96% selectivity with a space velocity increased from 52 h^−1^ to 230 h^−1^. Ethanol conversion increased from 64% to 88% at 380 °C with an increase in ethanol concentration of 10 wt % to 90 wt %, while ethylene selectivity decreased from 94% to 86% at 380 °C with the same increase in ethanol concentration. The catalyst deactivated quickly after 400 h of stability testing with temperatures be between 410 and 430 °C. Although the modification of γ-Al_2_O_3_ has produced much more favorable results than the original catalyst, the reaction temperature is still high, considering the modern industrial standards. On the other hand, the stability of the modified catalyst is relatively high as compared to other catalysts currently being developed, lasting 400 before deactivating.

### 2.2. Modification of HZSM-5

The ability of HZSM-5 to catalyze the dehydration of ethanol to ethylene at lower temperatures (200 to 300 °C) has made it commercially valuable and promising for further improvements in efficiency. The acidity and porosity of zeolites play an important role in the ethanol transformation into hydrocarbons [17]. At 300 °C, HZSM-5 can reach an ethanol conversion level of 98% and 95% ethylene selectivity. The main disadvantage of HZSM-5 is its acidity, which reduces its stability and coking resistance. As a solution, Zhang *et al.* (2008) and Ramesh *et al.* (2009, 2010, 2012) found that modification with phosphorous to reduce acidity maintained high ethylene selectivity (99.8%) while also increasing anti-coking ability [18,19,20,21]. These results were a trade-off with reaction temperature, as temperatures at or above 300 °C were necessary. Lanthanum modification was reported by Mao *et al.* (1989) and Ouyang *et al.* (2009) to improve catalytic ability at low temperatures [22,23]. Thus, Zhan *et al.* (2010) investigated the properties of lanthanum-phosphorous modified HZSM-5 [13]. Commercial HZSM-5 zeolite was impregnated with H_3_PO_4_ and La(NO_3_)_3_, stirred and evaporated, then dried and calcined to yield La-P-HZSM-5 of different compositions. The most favorable of the compositions was 0.5% La-2% P-HZSM-5, which reached 99.9% ethylene selectivity with 100% ethanol conversion at 240 °C. Moreover, the catalyst was able to maintain ethylene selectivity above 97% after 72 h, although no information was reported on its stability for longer periods of time. The catalyst most likely has a long life span, since La-P-HZSM-5 had a life span of 830 h before losing reactivity in the study done by Ouyang *et al.* (2009), but the inclusion of phosphorus could reduce the life span [23]. TG/DTG/DTA analysis showed restricted coke formation, as expected with the addition of lanthanum.

By modifying commercial catalyst HZSM-5, Zhan *et al.* (2010) managed to produce ideal data for the catalysis of ethanol dehydration to ethylene [13]. With almost 100% ethylene selectivity and ethanol conversion and low temperatures of 240 °C, 0.5% La-2% P-HZSM-5 is currently one of the most promising catalysts for industrial use. On top of achieving such high catalytic performance, the modifications made were inexpensive, another advantage to using 0.5% La-2% P-HZSM-5. More information on long term stability of the catalyst and effects of different reaction conditions is necessary before it can be employed in large scale plants.

Another modification developed by Guo *et al.* is nanoscale HZSM-5 zeolite catalysts [24,25,26,27]. The nanoscale HZSM-5 zeolite catalyst labeled as nano-CAT was obtained. The microscale HZSM-5 zeolite powder (crystal size: 1–3 μm) was commercially available [25]. By modifying commercial catalyst HZSM-5, Sendesi *et al.* (2012) has found that modification with Si/Al ratio. Based on TPR results, increasing Si/Al ratio from 25 to 250 was improved the redox properties, which will favor the speed of electron transfer [28]. Compared to conventional-sized HZSM-5 zeolites, the nanocatalysts reached 99.7% ethylene selectivity and 100% ethanol conversion at 240 °C, and maintains both ethanol conversion and ethylene selectivity above 98% for 630 h of reaction, exhibiting high stability. On top of that, the catalyst demonstrated good coking resistance. The nanoscale HZSM-5 appears to be the most ideal catalyst out of the current ones being studied, but before applying it in industry, the feasibility of scaling nanoscale catalysts to large commercial scale production plants while retaining laboratory results should be considered.

### 2.3. Heteropolyacids

Heteropolyacid salts have attracted attention as potential catalysts due to the multifunctional properties of the salt formation. Particularly, Ag_3_PW_12_O_40_ has demonstrated high catalytic ability, making it a promising catalyst for the dehydration of ethanol to ethylene, but its high acidity reduces its stability. Gurgul *et al.* (2011) investigated the influence of surface composition of Ag_3_PW_12_O_40_ on its catalytic ability and stability, especially observing the effects of air humidity [29]. The catalyst was prepared by reacting silver nitrate with tungstophosphoric acid, and the resulting salt was dried. From the catalytic tests, Ag_3_PW_12_O_40_ was found to achieve 99.8% ethylene selectivity at 2% humidity and 220 °C, but only about 70% ethanol conversion. At 9% humidity, ethylene selectivity was lowered to 99.2% at 220 °C, but ethanol conversion increased to almost 100%. Thus, Gurgul *et al.* (2011) concluded that the presence of water stabilized the surface composition of AgPW salt (e.g., Ag_3_PW_12_O_40_·3H_2_O) [29]. Long term stability of the catalyst was not detailed in the study, so whether the application of AgPW salt in industry is economically favorable or not is still left to be determined. However, the high ethylene selectivity (99.2%) at a low reaction temperature of 220 °C suggests that areas of the world with an average relative humidity of 9% should investigate the catalyst further, since the reaction temperature is lower than most of the current catalysts being studied. The lattice parameter for Ag_3_PW_12_O_40_·3H_2_O structure at 473 K was estimated to 11.93 Å [29]. It was also observed when the structure of H_3_PW_12_O_40_·6H_2_O with 12.15 Å [30].

Ciftci *et al.* (2012) also studied the modification of tungstophosphoric acid (TPA) by impregnating it into MCM-41 [31]. The resulting TPA-MCM-41 catalyst achieved 99.9% ethylene selectivity at 300 °C, with about 98% ethanol conversion. This far outperforms the results of pure TPA reported by Varisli *et al.* (2007) with only 77% ethylene selectivity at 250 °C [32]. Activities of silica supported tungstophosphoric acid (TPA), and salts of TPA were tested for the dehydration of ethanol [31]. TPA incorporated silicate structured new mesoporous catalysts were synthesized following one-pot hydrothermal and impregnation procedures. Surface area of TPA-MCM-41, which was prepared by impregnating TPA into MCM-41, was two orders of magnitude higher than the surface area of pure TPA and this catalyst showed very high activity in dehydration reactions of both ethanol and methanol [32]. Very stable ethanol conversion data near 100% was also reported, but no quantitative data on the long term stability was included. The use of TPA-MCM-41 may be more advantageous for areas without enough humidity for AgPW salt in terms of heteropolyacids, but the reaction temperature of 300 °C makes TPA-MCM-41 less favorable than catalysts such as 0.5% La-2% P-HZSM-5.

Varisli *et al.* (2008) studied the impregnation of silicotungstic acid in MCM-41, and found that the catalyst had 99.9% ethylene selectivity with about 99% ethanol conversion at 250 °C [33]. Due to higher surface area, it showed higher activity than TPA impregnated MCM-41. However, no data on the life span or long term stability of the catalyst was reported, so the catalyst should be further studied before it is considered for industrial application.

A W-Silicate-based heteropolyacid catalyst (TRC-92) developed by Varisli *et al.* (2010) showed 99% ethylene selectivity at 280 °C, but only about 70% ethanol conversion [34]. The W-Silicate-basedcatalyst (TRC-92), modified version of the hydrothermal synthesis procedures for the synthesis of vanadium, palladium, nickel and tungsten incorporated silicate structured mesoporous catalysts [34]. Despite the relatively low ethanol conversion though, the catalyst is capable of liquid phase reactions, because of the high stability of the solid catalyst and its having both meso and macropores. Although the low ethanol conversion may appear disadvantageous, the elimination of the need to vaporize ethanol before feeding it to the reactor may prove even more cost-efficient than 100% ethanol conversion.

A summary of the catalysts is listed in Table 2, along with a currently used industrial catalyst, SynDol, for a basis of comparison. SynDol catalyst based on MgO–Al_2_O_3_/SiO_2_ developed by Halcon SD has been applied commercially [35].

**Table 2:** Summary of catalysts for the dehydration of ethanol to ethylene and their catalytic ability.

Catalyst	Max ethylene selectivity	Ethanol conversion	Reaction Temperature	LHSV ^a^/ WHSV ^b^/ GHSV ^c^	Lifespan, Stability	Comments	Reference
TiO_2_/γ-Al_2_O_3_	99.4%	100%	360–500°C	26–234 h^−1 a^	400 h, stable	Lab modified	[15]
0.5% La-2% P-HZSM-5	99.9%	100%	240–280°C	2 h^−1 b^	Very stable	Lab modified	[13]
Nano-CAT	99.7%	100%	240°C	1 h^−1 b^	630 h, very stable	Lab modified	[25]
Ag_3_PW_12_O_40_	99.2%	100%	220°C	6000 h^−1 c^	Stable in 9% humidity	Lab synthesized	[29]
TPA-MCM-41	99.9%	98%	300°C	2.9 h^−1 b^	Very stable	Lab modified	[31]
STA-MCM-41	99.9%	99%	250°C	2.9 h^−1 b^	Stable	Lab modified	[33]
TRC-92	99.0%	70%	280°C	2.9 h^−1 b^	Very stable	Lab synthesized	[34]
SynDol (Halcon) (SD, USA)	96.8%	99%	450 °C	26–234 h^−1 a^	Very stable	Commercial catalyst	[15]

^a^ liquid hourly space velocity (LHSV); ^b^ weight hourly space velocity (WHSV); ^c^ gas hourly space velocity (GHSV).

## 3. Reaction Conditions

Many studies included data on the effects of changing certain reaction conditions on catalytic ability (summarized in Table 3 and Table 4), but it cannot be assumed that the effects apply to any catalyst of the ethanol dehydration reaction. For example, higher space velocity appears to lower ethanol conversion and ethylene selectivity, according to studies done on TiO_2_/γ-Al_2_O_3_ and SAPO catalysts by Chen *et al*. (2007), and Chen *et al*. (2010), but higher space velocity slightly increased ethylene selectivity for La-HZSM-5 in the study by Ouyang *et al*. (2009) [8,15,23]. Other changes in reaction conditions include the presence of water in the reactor in the form of humidity, which stabilizes AgPW catalyst but reduces ethylene selectivity in the study by Gurgul et al. that compared catalytic ability under 9% humidity with 2% humidity (2011) [29]. Matachowski *et al*. (2012) also reported higher stability of AgPW catalyst in 10% humidity, but showed results for higher ethylene selectivity and higher ethanol conversion [36]. Wang *et al.* (2011) found that the optimal ethanol feed mass fraction for Zn-Mn-Co-HZSM-5 was actually 34.4% while Petroleum Processing and Petrochemicals (2010) reported an optimal ethanol feed mass fraction of 74% for La-P-HZSM-5, significantly different values despite both catalysts being modified versions of HZSM-5 [37,38]. However, the two optimization studies and two humidity studies provide insights into whether or not water in the ethanol feed deactivates catalysts, and whether facilities must be added to remove water before the ethanol can be fed into the reactors. For all catalysts reviewed, increasing temperature increases ethylene selectivity up to an optimal reaction temperature, after which increasing the temperature reduces selectivity and stability of the catalyst.

**Table 3:** Effect of space velocity on catalytic ability of ethanol dehydration catalysts.

Catalyst	Reaction Condition	Condition Setting	Ethanol Conversion	Ethylene Selectivity	Reference
TiO_2_/γ-Al_2_O_3_	Space velocity (LHSV)	52 h ^−1^	100%	98%	[15]
		234 h ^−1^	96%	97%	
SAPO	Space velocity (WHSV)	2 h ^−1^	100%	100%	[8]
		30 h ^−1^	65%	20%	
La-HZSM-5	Space velocity (LHSV)	0.5 h ^−1^	100%	97%	[23]
		25 h ^−1^	39%	100%	

**Table 4:** Effect of humidity on catalytic ability of AgPW catalyst.

Catalyst	Reaction Condition	Condition Setting	Ethanol Conversion	Ethylene Selectivity	GHSV	Reference
AgPW	Humidity	2%	70% (470 K)	100% (470 K)	6000 h^−1^	[36]
		10%	100% (470 K)	80% (470 K)		
AgPW	Humidity	2%	75% (493 K)	100% (493 K)	6000 h^−1^	[29]
		9%	100% (493 K)	99% (493 K)		

## 4. Industrial Concerns

In addition to research on optimizing the catalysis of ethanol dehydration, investigations in improving other aspects of producing green ethylene have also made progress. Instead of conventional methods for obtaining ethanol from fossil fuels or by synthetic gas or even newer methods such as using corn feedstock, Algenol (USA) has proposed to produce ethanol by fermentation in algae [39]. In comparison to yeast fermentation, ethanol produced by algae can be removed without killing the algae. Because algae can continuously grow and produce ethanol without being killed, the need for regeneration of the algae is eliminated, as opposed to the need to regularly replenish yeast. As a result, Algenol researchers reported that by using algae, they could produce 6000 gallons of ethanol per acre per year, a significant increase from the 400 gallons per acre per year produced by corn production. However, since Algenol is only concerned with the production of ethanol and not ethylene, a link between the production of ethanol by algae and the rest of an ethylene processing plant must be considered, whether it is storage and transport of ethanol between two separate facilities or direct feed of ethanol into the reactor.

Chen *et al.* (2007) researched the application of microscale reactors instead of conventional fixed bed reactors and found that the smaller reaction systems increased surface-to-volume ratios, mass and heat transfer capabilities, process safety, yield, and efficiency [40]. Selectivity for ethylene increased from 98.9% to 99.3% for TiO_2_/γ-Al_2_O_3_ under identical reaction conditions, and ethylene yield increased from 0.67 g/(g-catalyst.h) to 72.7 g/(g-catalyst.h). Scaling miniature reactors to industrial proportions poses a problem, as even the slightest increase of size of the reactors will produce less favorable results, and increasing the quantity of microreactors to meet commercial demands is potentially more costly than simply using conventional fixed bed reactors.

Several other challenges must be overcome before the dehydration of ethanol to ethylene can replace steam-cracking fossil fuels to produce ethylene. Besides finding a reliable, renewable source of ethanol and developing ideal catalysts for the reaction, industrial concerns such as production cost, energy cost, catalyst regeneration, and most importantly, yield must be considered. Production costs may be lowered by increasing the space velocity of the plant, but doing so increases the optimal temperature and decreases the stability of the catalyst. An alternative is reducing the amount of equipment and machinery needed in the plant, which would also reduce the number of employees needed to operate. For energy costs, reducing reaction temperature while still achieving high selectivity is a priority, but the exiting fluid can also be used as heating fluid to recover heat and save energy. The exiting fluid is predominantly ethylene, diethyl ether, and water, and depending on the catalyst, the water may be directly fed back into the reactor with ethanol to maximize heat transfer from water. The diethyl ether may be converted to ethylene, but that would require another facility. Instead, it can be used as a fuel to reduce energy costs as well. Catalysts with long life spans would reduce the number of times they would need to be regenerated. With catalysts such as SynDol or nanoscale HZSM-5, which have long lifespans compared to other catalysts, separate equipment for catalyst regeneration may not be necessary. This would significantly reduce production costs as well.

If the currently researched technology were applied to an industrial plant while maintaining experimental results, the plant would be able to produce as much ethylene as steam-cracking plants, except at a steep cost of farmland. The Braskem ethanol to ethylene plant, currently the only commercialized ethanol to ethylene plant, imports 462 million liters of ethanol to produce 200,000 tons of ethylene per year [14]. To produce as much ethylene as the tenth largest ethylene complex, Equate Petrochemical Company’s plant (Kuwait), the plant would have to produce 1,650,000 tons of ethylene per year [5]. At 99.7% ethylene selectivity and 100% ethanol conversion for nanoscale HZSM-5, approximately the same amount of ethanol would be consumed. Using the density of ethanol at 20 °C, the following conversion can be made:

(1) $$16,500\text{ tons }\times \frac{1000\text{ kg}}{1\text{ ton}}\times \frac{1\text{ L}}{0.7893\text{ kg}}\times \frac{1\text{ gallon}}{3.7854\text{ L}}=552,200,000\text{ gallons}$$

Algenol’s technology of producing ethanol by algae fermentation can supposedly produce 6000 gallons of ethanol per acre per year [39]. This means:

(2) $$552,200,000\text{ gallons}\times \frac{1\text{ acre}}{6000\text{ gallins}}=92,000\;acres=370\;{\text{Km}}^{2}\;$$

To put this in perspective, all of the U.S. Virgin Islands or a third of Hong Kong would have to be used for algae farms. This is just to compete with the tenth largest ethylene plant. To produce 141 million tons of ethylene, the projected global demand for 2012, 32,000 km^2^ would have to be used. That is, an area around the size of Taiwan would be used on algae farms. A more feasible solution would be to use a mixed source ethanol feedstock—some produced by algae and some by other biomass. This way the amount of additional land needed for algae farms would be reduced, and the amount of corn or sugar cane used to produce ethanol would not cause competition with other industries, such as the food industry.

Of course, production and energy costs would have to be considered as well, in order to compare with steam cracking plants. The costs of constructing the algae facilities, transporting ethanol to the ethylene plant, purchasing or synthesizing catalysts in bulk, and heating the reactor to optimal reaction temperature are just a few of the many investments that would have to be made. A plant that produces 500,000 tons of ethylene per year would require 821,000 tons of ethanol, 22,000 tons of fuel, and a capital cost of $150 million (compare with $700 million for a cracking plant), according to Seddon [41]. The production cost of ethylene would depend mainly on ethanol prices, and currently ethanol prices are at about $910 per ton, as reported by Businessweek [42]. The International Renewable Energy Agency (IRENA) reported ethylene production costs at about $2000 per ton from corn feedstock in the U.S. ($1200 per ton from sugar cane feedstock, which is what the Braskem plant uses), while petrochemical ethylene only costs $600 to $1300 per ton [43]. Compared with the bioethylene production costs with the $1650 per ton price of ethylene reported by PRNewswire, the bioethylene plant would not gain any revenue [44]. However, using the algae technology instead of corn feedstock and more efficient catalysts like nano-HZSM-5 would significantly reduce production costs, and may make a bioethylene plant profitable. Nanoscale and microscale HZSM-5 zeolites, the crystal size of nanoscale HZSM-5 is in the range of 50–100 nm and that of microscale HZSM-5 is in the range of 1–3 μm [25]. In addition, the high demand for ethylene and increasing public awareness of green alternatives ensures that there is a market for green ethylene, with approximately 80% of the ethylene produced by the Braskem plant was sold before its construction [14]. The bioethylene produced may also be sold with a green premium to increase profits.

High energy costs account for much of the production costs as well, and a comparative study of different ethanol to ethylene processes by Arvidsson *et al.* (2011) includes an analysis on heat recovery and conservation to potentially reduce those costs [45]. Based on a 200,000 ton ethylene capacity, simply combining an ethylene plant and an ethanol plant into one refinery instead of using stand-alone plants would reduce the minimum hot utility demand from 130.9 MW to 79.2 MW. The minimum cold utility demand would also be reduced from 195.7 MW to 141.1 MW. In addition, the integration of flue gas with the ethylene reactors of the combined bio-refinery would further reduce the utility demands to 68.0 MW (hot) and 140.4 MW (cold). Alternatively, introducing mechanical vapor recompression on the rectifier distillate was predicted to have a hot utility demand of 32.1 MW, and a cold utility demand of 102.4 MW. Lastly, the delivery of high pressure (41 bar) steam to the chemical cluster resulted in a calculated hot utility demand of 76.0 MW and a cold utility demand of 137.6 MW. A summary of results is shown in Table 5. The application of any of the theoretical biorefinery configurations would greatly reduce energy costs, with the incorporation of mechanical vapor recompression being the most efficient. In combination with the algae technology and nanoscale HZSM-5, converting ethanol to ethylene may soon become a cost-effective reality.

**Table 5:** Comparison of ethanol to ethylene plant configurations [45].

Configuration	Minimum hot utility (MW)	Minimum cold utility (MW)	Net electricity (MW)	Net fuel (MW)
Stand-alone EtOH	112.2	147.6	24.3	0.0
Stand-alone Ethylene	18.7	48.1	−4.4	−15.9
Biorefinery	79.2	141.1	8.0	−7.9
Bio-F ^1^	68.0	140.4	8.5	7.5
Bio-MVR ^2^	32.1	102.4	−15.8	−7.9
Bio-VHP ^3^	76.0	137.6	17.1	−7.9

^1^ Biorefinery—Flue gas integration with ethylene reactors; ^2^ Biorefinery—Introduction of mechanical vapor recompression on the rectifier distillate; ^3^ Biorefinery—Very high pressure steam 41 bar (absolute pressure) steam delivery to the chemical cluster.

## 5. Conclusions

Despite much advancement being made in producing ethylene from ethanol, the process is not ready to replace fossil fuel methods in meeting the world demand for ethylene. Recent successes in increasing ethylene yield and lowering reaction temperature by modifying catalysts have revealed a number of catalysts that could be applied to industry, the most favorable one being nanoscale HZSM-5, which has a 99% ethylene yield at 240 °C and a lifespan of 630 h before ethylene selectivity decreased to below 98%. However, even with the development of catalysts such as Ag_3_PW_12_O_40_ and nano-HZSM-5 achieving over 99% ethylene yield at temperatures as low as 220 °C, and algae and microreactor technology potentially reducing other production costs, bioethylene is still not profitable enough to produce industrially. Recent findings on lowering energy costs have also appeared to make ethanol dehydration more profitable, but since they were based on a 200,000 tons capacity, a larger capacity plant may not save as much on energy expenditures. As the most widely produced organic chemical in the world, ethylene is produced at the rate of over 100 million tons per year. Until ethanol dehydration plants can achieve such high yield at costs competitive with steam-cracking plants, industry will continue to use the limited fossil fuels. Thus, future researchers should conduct their research in the context of economical factors and with respect to the other steps in the process of producing ethylene from ethanol. Studies focused on the catalysis of ethanol dehydration should keep catalyst stability and lifespan in mind and should shift goals towards lowering reaction temperature instead of increasing yield, and studies on ethanol production or reactor technology should not neglect the problem of linking parts of the ethylene production chain together. In addition, studies on catalysts should include information on the effects of changing various different reaction conditions, rather than just one or two. A reaction condition that no study addressed is the effect of using impure ethanol as a feedstock. This is particularly relevant to industry, because different ethanol feedstock sources contain different impurities, which may affect a certain catalyst’s catalytic ability or life span in different ways. Increasing the number of studies on algae technology may have the benefit of possibly increasing the yield of ethanol per acre of algae, and more studies on heat conservation in reactors may reduce energy expenditures needed for plant operation. Of course, one also considers simultaneously the transformation of ethylene (or ethanol) to alkanes or alkenes. It will produce products that are more valuable.
